# Elevated mutation rates are unlikely to evolve in sexual species, not even under rapid environmental change

**DOI:** 10.1186/s12862-019-1494-0

**Published:** 2019-08-28

**Authors:** Daniel Romero-Mujalli, Florian Jeltsch, Ralph Tiedemann

**Affiliations:** 1 University of Potsdam, Evolutionary Biology/Systematic Zoology, Karl-Liebknecht-Strasse 24-25, 14476 Potsdam, Germany; 2 Plant Ecology and Nature Conservation, University of Potsdam, Am Mühlenberg 3, 14476 Potsdam, Germany; 3 Berlin-Brandenburg Institute of Advanced Biodiversity Research (BBIB), 14195 Berlin, Germany

**Keywords:** Individual-based models, Mutation rate, Mutator locus, Directional climate change, Recombination, Beneficial mutations, sexual species

## Abstract

**Background:**

Organisms are expected to respond to changing environmental conditions through local adaptation, range shift or local extinction. The process of local adaptation can occur by genetic changes or phenotypic plasticity, and becomes especially relevant when dispersal abilities or possibilities are somehow constrained. For genetic changes to occur, mutations are the ultimate source of variation and the mutation rate in terms of a mutator locus can be subject to evolutionary change. Recent findings suggest that the evolution of the mutation rate in a sexual species can advance invasion speed and promote adaptation to novel environmental conditions. Following this idea, this work uses an individual-based model approach to investigate if the mutation rate can also evolve in a sexual species experiencing different conditions of directional climate change, under different scenarios of colored stochastic environmental noise, probability of recombination and of beneficial mutations. The color of the noise mimicked investigating the evolutionary dynamics of the mutation rate in different habitats.

**Results:**

The results suggest that the mutation rate in a sexual species experiencing directional climate change scenarios can evolve and reach relatively high values mainly under conditions of complete linkage of the mutator locus and the adaptation locus. In contrast, when they are unlinked, the mutation rate can slightly increase only under scenarios where at least 50% of arising mutations are beneficial and the rate of environmental change is relatively fast. This result is robust under different scenarios of stochastic environmental noise, which supports the observation of no systematic variation in the mutation rate among organisms experiencing different habitats.

**Conclusions:**

Given that 50% beneficial mutations may be an unrealistic assumption, and that recombination is ubiquitous in sexual species, the evolution of an elevated mutation rate in a sexual species experiencing directional climate change might be rather unlikely. Furthermore, when the percentage of beneficial mutations and the population size are small, sexual species (especially multicellular ones) producing few offspring may be expected to react to changing environments not by adaptive genetic change, but mainly through plasticity. Without the ability for a plastic response, such species may become – at least locally – extinct.

**Electronic supplementary material:**

The online version of this article (10.1186/s12862-019-1494-0) contains supplementary material, which is available to authorized users.

## Background

Local adaptation to changing environmental conditions, such as directional climate change, becomes of high importance for organisms with limited dispersal abilities, or when physical barriers preventing dispersal are present.

In such scenarios, organisms can adapt by genetic changes and / or phenotypic plasticity. For genetic changes to occur, mutations are the ultimate source of novel variation, and it is generally assumed that a mutation is a rare event [1]. Consequently, individual-based models of explicit genetics typically assume small and constant (i.e., non-evolving) mutation rates [2–4]. According to evidence, only few mutations are adaptive; many deleterious; and some are neutral [5]. There is however genetic variation in DNA repair and replication processes [6–9], affecting the probability of a mutation to occur. Genetic loci affecting the origin of new variation (i.e., the mutation rate) have been termed “mutator” loci. They may be subject to selection, and selective forces may depend on the environmental context or scenario.

Previous work investigating the evolution of the mutation rate has found that the fate of mutator alleles may differ for sexual and asexual organisms [10, 11]. In asexual organisms mutator alleles are associated with the mutations they caused, thus mutator alleles leading to an increase of the mutation rate can increase in frequency by hitchhiking with beneficial mutations at other loci [12]. In contrast, in sexual organisms given that recombination breaks linkage disequilibrium, the mutator allele will be separated soon from a beneficial mutation it has caused and will not hitchhike to high frequency [10]. Consequently, the evolution of mutation rate for a sexual organism is rather unlikely, and the mutation rate is then expected to stay close to a minimum achievable, limited either, by the costs of replication fidelity or by the drift limit [13, 14].

Contrary to previous authors, a recent simulation study suggests that the mutation rate can evolve to relatively high values also in sexual species and this can advance invasion speed and promote adaptation to novel environmental conditions along an environmental gradient [15]. The proposed mechanism is induced linkage disequilibrium between the dispersal locus and the mutator locus (both were evolving traits) that arise from spatial sorting and iterated founder event [15]. The same authors also found that these results still held under assumptions of 90% of the mutations being lethal.

The present work uses an individual-based modeling approach focused on local adaptation to test the evolution of the mutation rate in a population of a sexual species experiencing directional climate change, under different scenarios of linkage disequilibrium (unlinked to complete linkage). The aim was to investigate, following the findings of Cobben et al. [15], whether directional climate change scenarios can also lead to the evolution of an elevated mutation rate in a sexual species. This work employs an alternative method for the simulation of beneficial mutations, i.e., implementing a distribution of mutation effects inspired by the concept of slightly deleterious mutations [16, 17]. Furthermore, different scenarios of environmental stochasticity or noise color were investigated to test whether the mutation rate could vary among organisms experiencing different habitats.

## Results

**Fig. 1:**
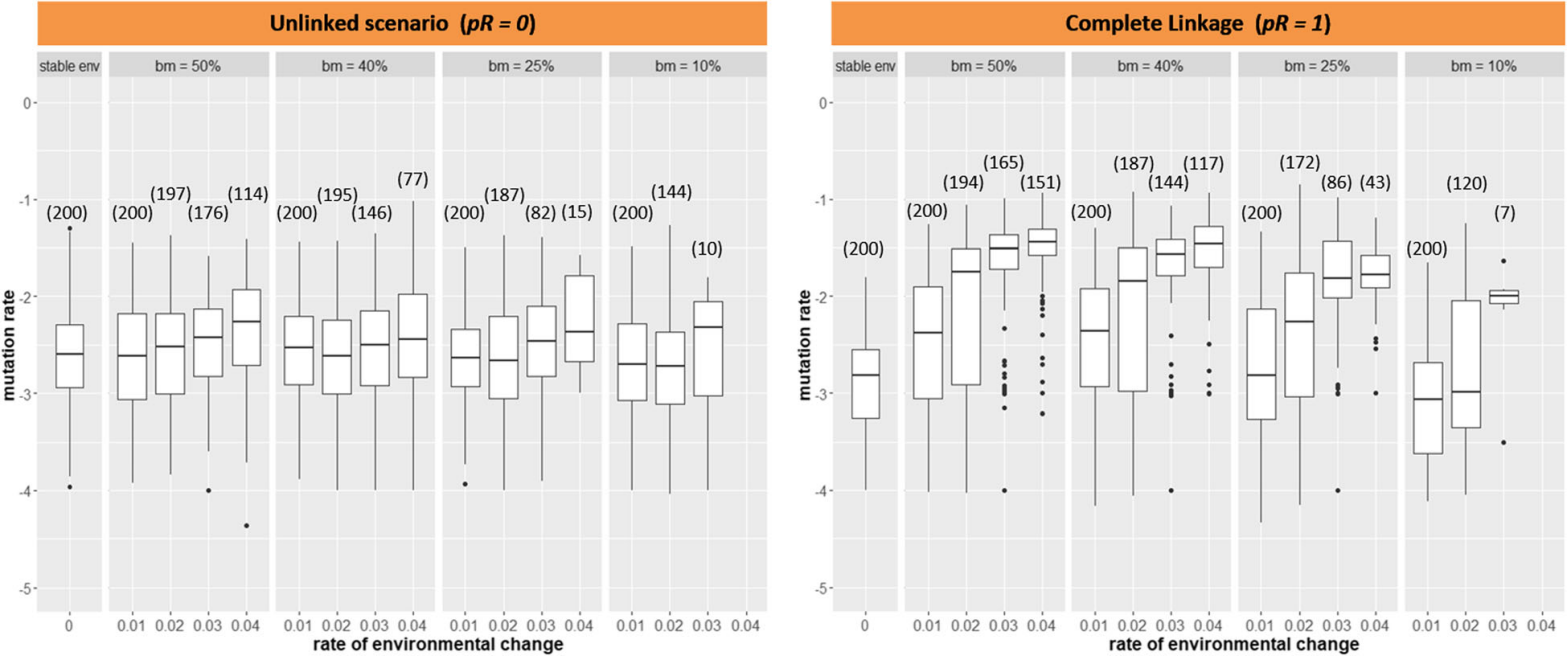
The evolution of the mutation rate under scenarios of directional environmental change, probability of recombination *pR*, and percentage of beneficial mutations *bm*. Each data point corresponds to the mean mutation rate present in the population at the end of each simulation run (200 generations). The number of data points per box is shown in parenthesis (selection parameter *γ = 2.2*). A weaker selection regime (*γ = 3.2*) showed a similar pattern (data not shown). Stable env: stable environment (no directional environmental change)

In our simulations, the mutation rate followed different evolutionary trajectories, relative to the percentage of beneficial mutation *bm* and the probability of recombination *pR*: Overall, the evolved mutation rate reached higher values when increasing the percentage of beneficial mutations *bm*, and this was independent of scenarios of probability of recombination *pR* and rate of environmental change (Fig. 1, Additional file 1: Figure S2). The mutation rate evolved to relatively high values mainly under scenarios of complete linkage (*pR = 1*), relatively rapid directional climate change, and 25% or higher percentage of beneficial mutations (Fig. 1). Under the unlinked recombination scenario (*pR = 0*), the mutation rate evolved to relatively high values only if the rate of environmental change was fast (*η = 0.04*) and the percentage of beneficial mutations was high (50%; Fig. 1). An intermediate recombination rate (*pR = 0.5*) yielded intermediate results (Additional file 1: Figure S1).

**Fig. 2:**
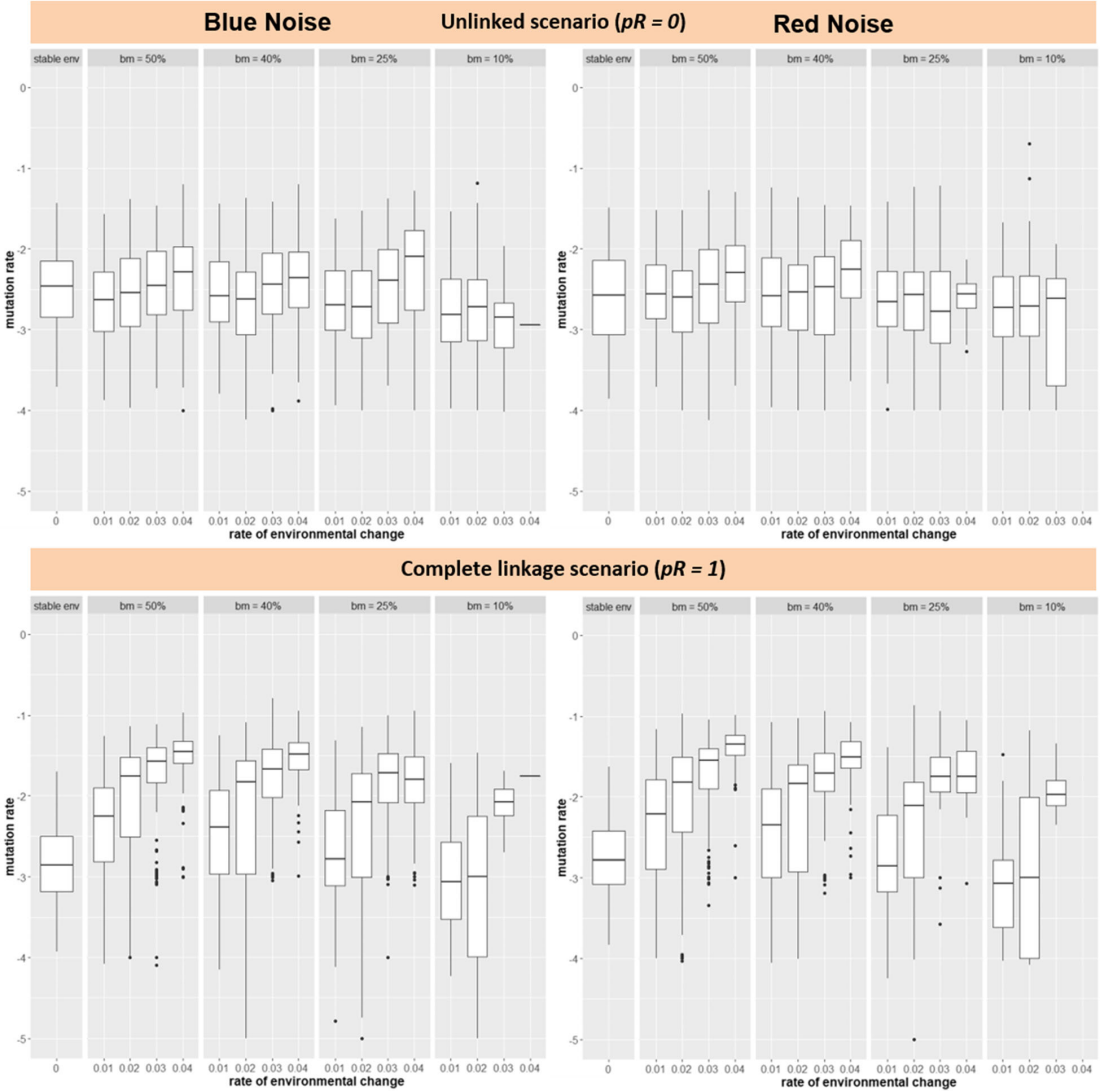
The evolution of the mutation rate under scenarios of blue (left) and red (right) noise, directional environmental change, probability of recombination *pR*, and percentage of beneficial mutations *bm*. Each data point corresponds to the mean mutation rate present in the population at the end of each simulation run (200 generations). Stable env: stable environment (no directional environmental change)

In general terms, for a population of a sexual species, it seems unlikely for the mutation rate to evolve to higher values under directional climate change scenarios, unless the rate of change is very rapid (*η = 0.04*) and the percentage of beneficial mutations is high (50%). This result is robust under different scenarios of environmental stochasticity or noise color (Fig. 2).

## Discussion

The aim of this study was to investigate whether the mutation rate could evolve to higher values in the course of local adaptation of an isolated population of a sexual species experiencing directional climate change. This work was inspired by a previous study that found adaptive evolution of the mutation rate which advances invasion speed of a sexual population inhabiting an environmental gradient [15]. According to our results, the mutation rate can evolve to relatively high values only under conditions of complete linkage or, without such linkage, under the assumption of 50% beneficial mutations. Under complete linkage, the mutation rate can evolve under intermediate to high rates of environmental change, and when the percentage of beneficial mutations is at least 25%. The scenario of complete linkage suggests mutation rates can be expected to evolve in asexual organisms, which is in line with current theory [10, 11]. For example, it was demonstrated for *Escherichia coli* populations that mutator alleles could be fixed by hitchhiking on beneficial mutations at other loci [12]. In a sexual species, scenarios resembling intermediate to complete linkage may occur if the mutator locus is somehow linked to the trait under selection (e.g., by close proximity on the same chromosome), such that it can hitchhike on beneficial mutations occurring on the evolving trait. Scenarios of intermediate linkage (*pR = 0.5*) can also yield slightly elevated mutation rates, relative to the unlinked scenario. However, assuming a definite location of the mutator locus in the genome (as, e.g., a locus encoding for a DNA polymerase), most traits under selection can be expected to be encoded far away from the mutator locus, such that the unlinked scenario seems most appropriate for sexually reproducing species.

On the other hand, under unlinked recombination scenarios, the mutation rate evolved to relatively higher values only under scenarios of 50% beneficial mutations, particularly when the rate of environmental change was fast. When the percentage of beneficial mutations was low (10 and 25%), either there were too few data points [2] to derive any conclusion, or the population went extinct for all runs when the rate of environmental change was increased. Thus, the likelihood of beneficial mutations occurring under scenarios of rapid climate change becomes of high importance for organisms with limited plasticity and standing genetic variation, in order to allow for evolutionary rescue in specialist species. These results are in contrast with those in Cobben et al. [15] where the mutation rate facilitated range expansion and evolved towards higher values even under the assumption of 90% lethal mutations. In our model, given that we implemented the percentage of beneficial mutations *bm* directly into the distribution of fitness effects of mutations, the negative effects of mutations (especially when *bm* was low) became stronger, the higher the rate of directional environmental change. Under these conditions, elevated mutation rates only evolved in scenarios of high percentage of beneficial mutations. This emphasizes potential outcome differences in studies focused on the evolution of the mutation rate depending on the implementation methods for deleterious mutations. Since our method is consistent with the concept of slightly deleterious mutations [16, 17], our simulation of beneficial mutations is presumably more realistic than using a gaussian distribution centered on zero, typically for IBMs of explicit genetics (e.g., [2, 15, 18]) under scenarios of directional selection. According to empirical data, however, most mutations are negative, some are neutral, and only few are beneficial [5]. The common assumption in IBM eco-evolutionary models of 50% beneficial mutations is therefore unrealistic and may overestimate evolutionary rescue (or invasion speed) under directional climate change scenarios. We interpret our results as indication that implementation of beneficial mutation percentages more in line with reality may preclude evolution of elevated mutation rates in sexual species, unless there is strong linkage between mutator and trait locus.

According to our model, the evolution of the mutation rate may be possible in asexual unicellular organisms, provided that inherited differences in the function of repair and replication mechanisms exist which could serve as “mutator”. In sexual unicellular organisms the mutation rate can evolve if the organisms experience high rate of environmental change and the probability of occurring beneficial mutations is high, or if mutator genes happen to occur close to genome sequences coding for the evolving trait, such that they can hitchhike with beneficial mutations at other loci. In multicellular organisms, the mutator locus is expected to be present and affect germ and soma alike. This was not considered in our model and represents a model limitation. In these organisms an increase in genomic mutation rate may lead to tissue damage and reduced survival. In the model, the mutation rate affected only the process of inheritance of the traits into the next generations. Therefore, the mutation rate may still evolve in multicellular organisms as suggested by the model if the effects of increased mutation rates compromise survival after reproduction takes place [15], or unless the mutation rate can be somehow increased for segments of the genome (e.g., traits, locus, germ-line). The later scenario may not be consistent with the mutator locus approach as implemented here (e.g., a locus encoding for a DNA polymerase). However, the immune system of vertebrates is an example of high frequency mutations locally restricted to few genes and cell types in multicellular organisms and that has evolved in response to the constant need of novel mutations in the arm race between host and pathogens [14, 19].

Our findings that mutation rates evolve to higher values only with unrealistically high percentages of beneficial mutations or complete linkage seem to be independent from some characteristics of the environmental change, as it was replicated for the different levels of environmental stochasticity or noise color. This supports and expands the observation of no systematic variation in the mutation rate among asexual organisms experiencing different habitats [14, 20], to sexual species. Thus, the mutation rate is expected to be determined by “deep general forces” balancing the deleterious effects of mutations and the physiological cost of further reducing mutation rates [20], and not by external properties of the habitat. However, there are environmental conditions (e.g., chemicals, radiation) that produce DNA damage or modify the chemistry of enzymes potentially affecting replication fidelity and promoting mutations [21]. Such conditions lead to non-adaptive elevations of the mutation rate. Another potential source of variation in the mutation rate is the difference in condition between individuals and populations. Increased mutation rates could occur through costs of DNA repair in maladapted individuals/populations experiencing a lag-load to the moving environmental optimum under environmental change. It has been shown that environmental and genetic stress compromise DNA repair mechanisms and therefore, causes individuals to pass on a greater mutation load to their offspring [9, 22, 23]. The phenotypic condition can be directly related to the ability of individuals of overcoming the physiological cost of high-fidelity replication [8]. Thus, individuals able to pay the cost of fidelity are expected to reduce the mutation rate down to the drift limit, as further reductions will be effectively neutral [14]. Those not able to pay the cost are expected to experience relatively elevated values of the mutation rate [8, 14].

## Conclusions

In conclusion, considering that 50% beneficial mutations may be an unrealistic assumption, and that recombination is ubiquitous in a sexual species, results in this study suggest that, it is unlikely for the mutation rate to evolve to elevated values in a sexual species experiencing directional climate change scenarios. Instead, mutation rate will be under stabilizing selection at the minimal value allowed by limitations by costs of replication fidelity or limitations imposed by the drift limit, as already proposed in the literature [13]. Furthermore and in line with Drake’s observation [20], our results indicate that the mutation rate can be expected to be relatively similar among organisms experiencing different habitats. Though the frequency of beneficial mutations remains an elusive quantity [5], empirical estimations of beneficial mutations in *E. coli* seem to be far below the lowest scenario investigated in this model [24]. If this observation applies to sexual species as well, the evolution of the mutation rate towards elevated values under sexual recombination will become even less likely. It is important to consider that conclusions derived from this study apply under the assumption of a mutator locus affecting replication fidelity. The presence and action of other mechanisms affecting the mutation rate (e.g., epigenetics mechanisms) may affect the results as reported in this study [14, 19]. Therefore, when the percentage of beneficial mutations is small, and populations are not large enough, sexual species (especially multicellular ones) producing few offspring may be expected to buffer their ability for local adaptation mainly through standing genetic variation and plasticity, provided that movement opportunities are constrained. Future work should focus on understanding the potential role of standing genetic variation, polygenic selection, epigenetics and phenotypic plasticity in the ability for local adaptation of sexual species under scenarios of directional climate change and when the probability of beneficial mutations is low.

## Methods

In order to investigate the evolution of the mutation rate under directional climate change scenarios, we designed a spatially implicit individual-based model (IBM) of a panmictic diploid population of a sexual species with non-overlapping generations experiencing directional climate change (a trend of the mean climatic variable such as temperature). Our model is based on two previous IBMs: Björklund et al. [25] for the simulation of environmental scenarios (including noise color) and the density dependence effect on fecundity; and Cobben et al. [15], for the simulation of explicit genetics on the inheritance of mutator and adaptation loci. The population was assumed to be geographically isolated, thus no migration was possible, such that the focus was on local adaptation. The mutation rate could evolve, and different scenarios of linkage disequilibrium and beneficial mutations were considered.

### Environment

The environment was stochastic and defined an optimum mean phenotype *θ*_*t*_ that moved at constant speed per generation. This environmental scenario has been considered best suit to investigate the effect of climate change [18, 26]. Thus, *θ*_*t*_ *= θ*_*0*_ *+ η t* impose the directional climate trend, where *θ*_*0*_ *= 0* was the initial environmental optimum (when *t = 0*) and *η*, the rate of environmental change. The parameter *η* was changed to simulate different scenarios of environmental change (e.g., no change, slow, medium, rapid change).

Environmental stochasticity or noise color was implemented as follows: The parameter θ^*^_t_ *= θ*_*t*_ *+ ϕ*_*t*_, was the realized environmental state with noise *ϕ*_*t*_ *= αϕ*_*t-1*_ *+ βξ*_*t*_. The autocorrelation coefficient *α* indicated the level of environmental correlation and therefore the noise color: *− 1 < α < 0*, blue noise; *α = 0*, white noise, and *0 < α < 1*, red noise. Three scenarios of *α* were considered, based on Björklund et al. [25]: blue noise (*α = − 0.7*); white noise (*α = 0*), and red noise (*α = 0.7*). The parameter *β* determined the environmental variance, according to *β = σ* $\sqrt{1-{\alpha}^2}$, as in [27], where *σ*^*2*^ *= 1* was the environmental variance. The parameter *ξ*_*t*_ was a random value, normally distributed with zero mean and unity of variance.

### Population dynamics

ndividuals in the population were characterized by the following traits: sex, stage (whether adult or juvenile), phenotype *zi*, determined by the alleles at the adaptation locus, and a mutator locus whose alleles determine the genetic mutation rate. The phenotype and the mutator locus were considered evolving traits for the model. At the beginning of each simulation run, the population composed of 1000 individuals, at carrying capacity *K*, was assumed to be locally adapted. Therefore the alleles coding for the phenotype were initialized randomly from a normal distribution centered in *θ*_*0*_ and variance *V = VG / 2 L*, where *VG* is the initial genetic variance present in the population, and *L* the number of loci which was set to 1 for all runs in this study. The phenotype *z* of individual *i* was determined by additive effects of alleles at the adaptation locus. As in Cobben et al. [15] the mutation rate was given by *μ = 10*^*-exp*^, where *exp = (l*_*m,1*_ *+ l*_*m,2*_*) / 2*. The alleles at the mutator locus *l*_*m,1*_ and *l*_*m,2*_ were initialized randomly according to a discrete uniform distribution in range *[2; 4]*. Therefore, the model’s initial conditions already incorporated intraspecific variation in the mutation rate between individuals in the population (initial mean mutation rate of *10*^*− 3*^). Assuming initial intraspecific variation in the mutation rate facilitates its evolutionary dynamic and reduces an otherwise relatively high extinction risk during early simulation time.

### Degree of local adaptation

Each individual was tested on its ability for local adaptation given by the match of its phenotype *zi* to the current environmental optimum θ^*^_t_. Stabilizing selection was assumed according to:

$$ wi={e}^{\frac{-{\left( zi-{\theta}^{\ast }t\right)}^2}{2{\upgamma}^2}} $$

The variable *wi* indicates the degree of local adaptation of individual *i*, and *γ*, the strength of selection, affected the width of the fitness function, and was set to 2.2. This means that individuals having a phenotype departing from the optimum in *1SD* phenotypic units (*SD*: standard deviation) will have a fitness of 90% (relative to the maximum fitness) which would be moderate selection according to [25].

### Fecundity

The mating system was random mating, where females mated only once and males could repeat mating (lottery polygyny). The fecundity *λ* of the reproductive couple was the sum of the scaled degree of local adaptation values *w’i* after considering density dependence effects, as in Björklund et al. [25]:

$$ w^{\prime }i={wie}^{\left(1-\frac{N}{K}\right)} $$

where *N* is the population size and *K*, the carrying capacity. Each couple (partners *i*,*j*) produced a number of offspring randomly drawn from a Poisson distribution with expectancy *λ = w’i + w’j.*

### Inheritance

**Fig. 3:**
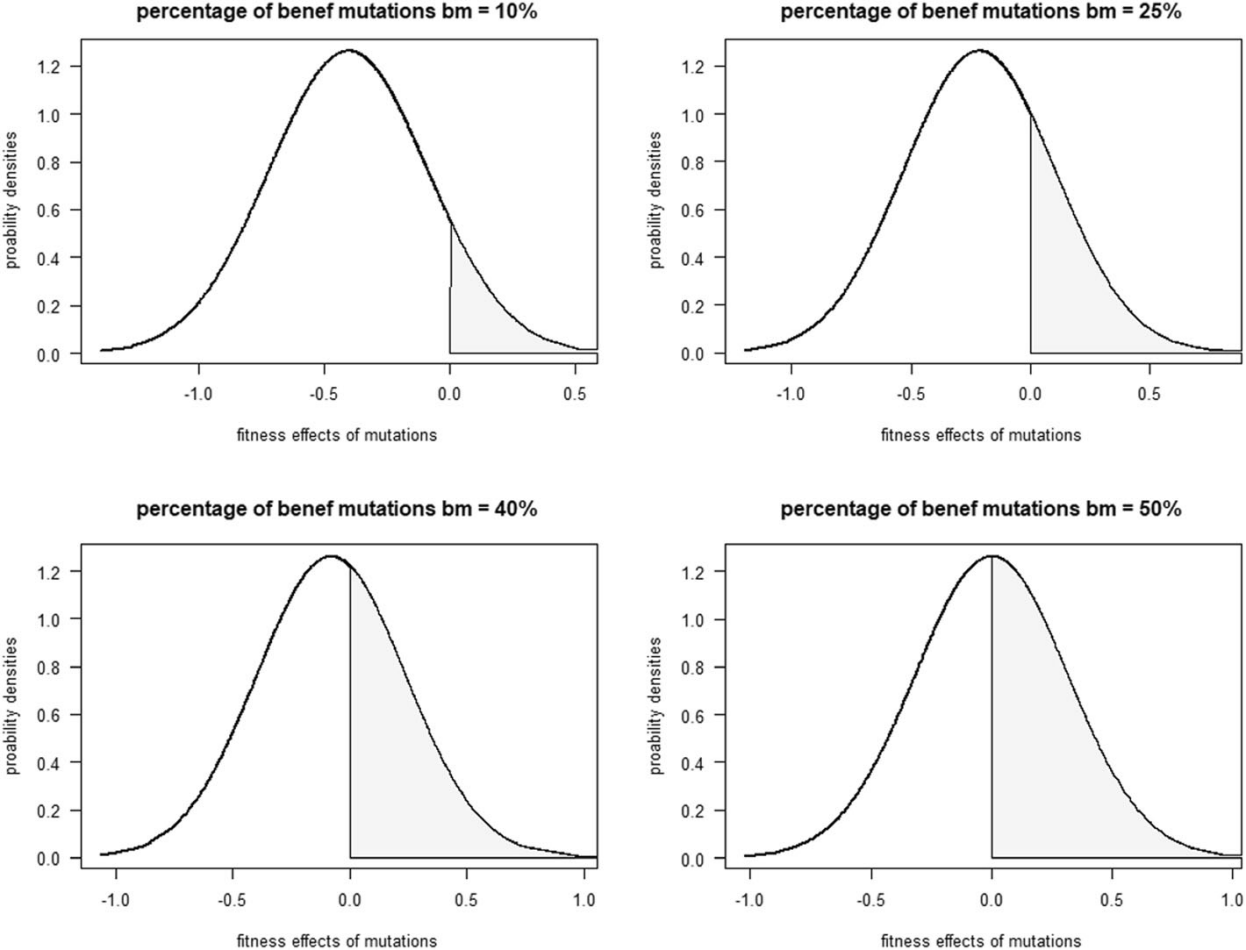
Distribution of fitness effects of mutations according to different scenarios of percentage of beneficial mutations *bm*. Beneficial mutations are shown in light grey color. Variance of the distribution *MV = 0.1*

Two scenarios of recombination were considered: unlinked and complete linkage (Table 1). During the process of inheritance, allele values for each locus (adaptation locus and mutator locus) of the inherited haplotype were picked randomly from the corresponding parental locus (unlinked scenario). In complete linkage scenario, alleles at the mutator locus were linked and migrated together with the corresponding alleles at the adaptation locus. For comparison, we also implemented an intermediate linkage (probability of recombination *pR = 0.5*). Then, mutations took place with probability *μ* determined by the alleles at the mutator locus as explained above. In case that a mutation occurred, its effect for the adaptation locus was randomly drawn from a normal distribution with zero mean and variance equals to the variance of the distribution of fitness effect size of mutations, which was an input parameter [18]. The assumption of a Gaussian distribution is consistent with analysis of mutation effects [28, 29]. In the model, the mean *x* of the distribution of mutation effect size could change according to the scenario of percentage of beneficial mutations (input parameter, Table 1). This applied for the adaptation locus only. This approach granted that beneficial mutations – mutations that pushed the trait in the direction of *θ*_*t*_ – actually occurred at different probabilities as shown in the distribution of mutations fitness effect size (Fig. 3). This can be important since under scenarios of directional selection the common assumption in individual-based models of explicit genetics of symmetric distribution of beneficial and deleterious mutations can overestimate the amount of arising beneficial mutations (e.g., [12, 17]). In fact, the shifted distributions approximates the mutational effects according to the model of slightly deleterious mutations [16, 17], albeit mutational effects under this model often exhibit skewed distributions. When a mutation occurred at the mutator locus, its effect consisted in adding a value randomly drawn from [− 1, 0, 1] to the mutated allele.

**Table 1:** Parameters values and description (values in parentheses were implemented to evaluate the robustness of outcomes)

Parameter	Value	Description
*K*	1000	Carrying capacity
*γ*	2.2 (3.2)	Strength of selection
*σ* ^*2*^	1	Variance of the stochastic environment
*θ*_*0*_	0	Initial environmental optimum
*η*	0, 0.01, 0.02, 0.03, 0.04	Rate of environmental change
*z*	Evolving trait	Ecological phenotype
*VG*	0.2	Initial genetic variance present in the population
*L*	Number of loci per evolving trait	1
*μ*	Evolving trait, range [0; 1]	Mutation rate
*MV*	0.2	Variance of the distribution of mutations fitness effect size
*bm*	10, 25, 40, 50	Percentage (%) of beneficial mutations
*pR*	0 (unlinked), (0.5), 1 (complete linkage),	Probability of recombination
*t*	300	Time limit per simulation, in generations. The last 200 generations were exposed to the treatment of directional climate change

After the process of inheritance, all adults died, and newborns took over the population (non-overlapping generations).

### Method for beneficial mutations

The mean *x* of the distribution of mutation effect size was given by *x = ε – y* $\sqrt{MV}$, where *ε = 0* was the Z-score cutting value between deleterious and beneficial mutations. The parameter *MV* was the variance of the distribution, and *y* was given by the quantile function of the normal distribution with probability *p = 1 – q*. The parameter *q* was the desired proportion of beneficial mutation (e.g., 0.25 for 25% scenario of beneficial mutations).

### Simulation experiments

To study the evolution of the mutation rate, different scenarios of rate of environmental change, stochastic noise color, probability of recombination, and percentage of beneficial mutations were implemented (Table 1). The scenarios of beneficial mutations were implemented only for the environmental conditions *η > 0* (directional climate change). For the stable environment (*η = 0*), the effect of mutations was drawn from a gaussian distribution centered in zero as it is commonly done in IBMs of explicit genetics [2, 4, 15] and acted as a control. Note that under directional environmental change, this is exactly our scenario of 50% beneficial mutations. Simulation experiments consisted of 200 replicates, lasting for 300 generations each: 100 generations of stable environment followed by 200 generations of treatment of directional climate change. Simulations were run under white stochastic environmental noise unless otherwise specified. The sequence of operations in the model were: update of the environmental optimum, degree of local adaptation, reproduction, mortality of adults, and update of phenotype of the new generation before repeating the loop. If extinctions occurred, the data was not used for the analysis.

The visualization of the data was done in r project v3.4.2 and the model was programmed in Netlogo v6.0.2.

## Additional file

Additional file 1: Additional supporting data. (DOCX 756 kb)

## Data Availability

All data generated or analyzed during this study are available at https://github.com/danielrm84?tab=repositories.

## Abbreviations

*bm*: Percentage of beneficial mutations
*exp*: Exponent value
IBM: Individual-based model
*K*: Carrying capacity
*L*: Number of loci
*l*_*m,1*_ and *l*_*m,2*_: Alleles at the mutator locus
*MV*: Variance of the distribution of mutations effects
*N*: Population size
*p*: Probability of non-beneficial mutations
*pR*: Probability of recombination;
*q*: Probability of beneficial mutations
*SD*: Standard deviation
stable env: Stable environment (no directional environmental change)
*t*: Time in generations
*V*: Phenotypic variance
*VG*: Initial genetic variance present in the population;
*w’i*: Scaled degree of local adaptation
*wi*: Degree of local adaptation (fitness proxy)
*x*: Mean of the distribution of mutations effects
*y*: Quantile function of the normal distribution
*zi*: Phenotype of the *i-*th individual
*α*: Autocorrelation coefficient
*β*: Adjusted environmental variance;
*γ*: Strength of selection
*ε*: Z-score cutting value between deleterious and beneficial mutations
*η*: Rate of directional environmental change
*θ*^***^_*t*_: Realized environmental state after considering stochasticity
*θ*_*0*_: Initial environmental optimum
*θt*: Optimum mean phenotype
*λ*: Fecundity
*μ*: Mutation rate
*ξ*_*t*_: Random normally distributed variable
*σ*^*2*^: Environmental variance
