# Supplementary material for: Elevated mutation rates are unlikely to evolve in sexual species, not even under rapid environmental change
Source: BMC Evol Biol. 2019 Aug 28;19:175. doi: 10.1186/s12862-019-1494-0 (PMC6714099; doi:10.1186/s12862-019-1494-0)
Supplement: Supplementary file 1 — Additional supporting data. (DOCX 756 kb) [file 12862_2019_1494_MOESM1_ESM.docx]

**SUPPLEMENTARY MATERIAL**

**
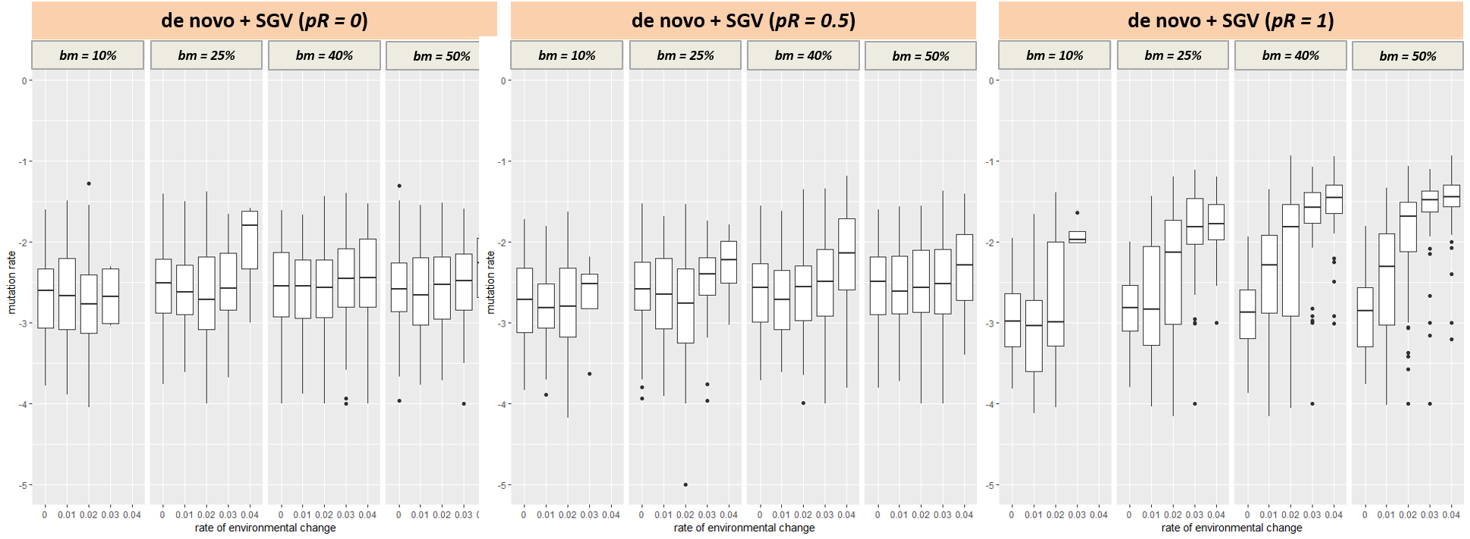
**

**Fig. S1** Evolution of the mutation rate per scenario of beneficial mutations *bm* and scenario of recombination: unlinked (*pR = 0*), intermediate (*pR = 0.5*), and complete linkage (*pR = 1*). Each data point corresponds to the mean mutation rate present in the population at the end of each simulation run (200 generations).


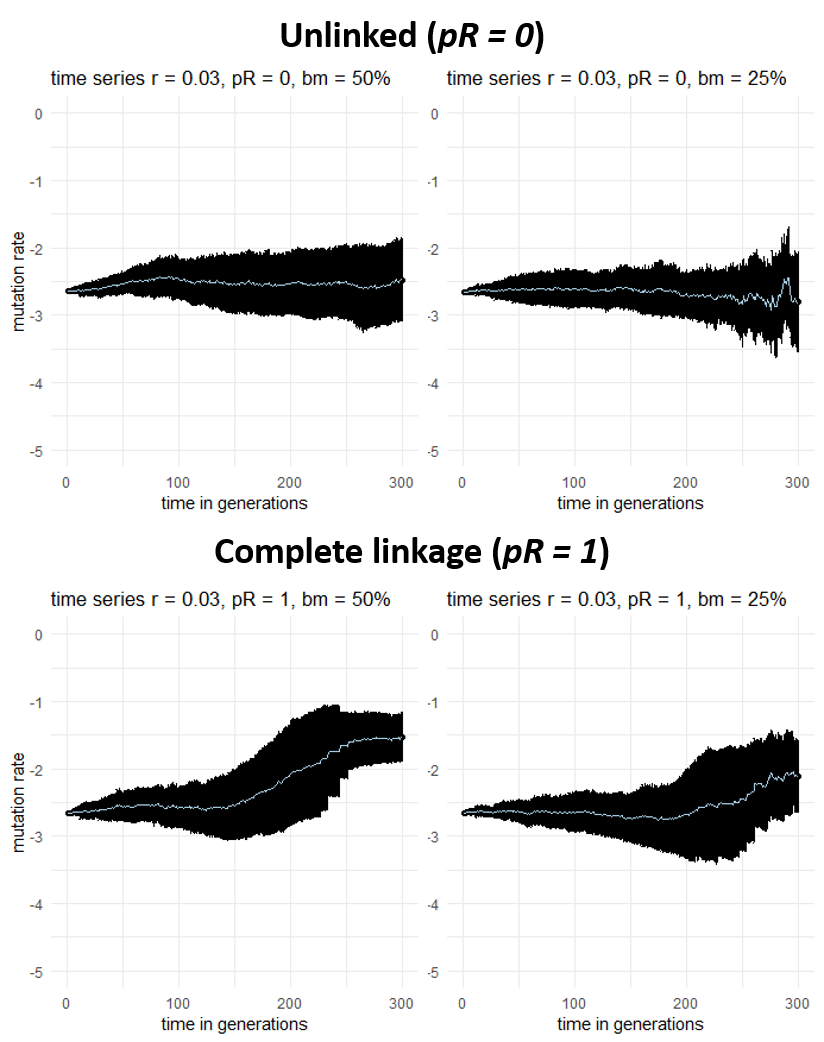


**Fig. S2** Trajectory (time series) of the evolutionary dynamics of the mutation rate (mean ± standard deviation from 30 replicates per condition) in a population experiencing rapid directional climate change (r = 0.03), under scenarios of unlinked (upper panel) and complete linkage (lower panel). *pR*: probability of recombination, and *bm*: percentage of beneficial mutations.
